# The Correlation Between the Natural Course, Pathologic Properties With Ki‐67 Expression in Lung Adenocarcinoma Presenting as Ground‐Glass Nodules

**DOI:** 10.1002/cam4.70390

**Published:** 2024-11-05

**Authors:** Shaohui Huang, Huanhuan Zhou, Chenchen Lin, Ziqi Wang, Lijun Shen, Ya Sun, Meihui Wei, Zhiwei Xu, Xiaoju Zhang

**Affiliations:** ^1^ Department of Respiratory and Critical Care Medicine Zhengzhou University People's Hospital, Henan Provincial People's Hospital Zhengzhou China; ^2^ Henan International Joint Laboratory of Diagnosis and Treatment for Pulmonary Nodules Zhengzhou China; ^3^ Xinxiang Medical University Xinxiang China

**Keywords:** ground‐glass nodules, Ki‐67, lung adenocarcinoma, natural course, volume doubling time

## Abstract

**Background:**

With the increasing use of lung cancer screening, the detection of ground glass nodules (GGNs) has risen. However, the natural course of GGNs and their relationship to pathologic features remains unclear. Differentiating between invasive and pre‐invasive lesions based on GGN growth may improve clinical intervention timing. Ki‐67, a proliferation marker, holds value in assessing tumor malignancy. This study analyzes the association between GGN growth, pathology, and Ki‐67 expression to provide new insights into early‐stage lung cancer management.

**Methods:**

We retrospectively evaluated 183 GGNs with at least two preoperative CT scans. Nodule location, type, natural course, and volume doubling time (VDT) were compared between invasive adenocarcinoma (IAC) and pre‐IAC groups. We also assessed differences in Ki‐67 expression and correlated VDT with Ki‐67 levels.

**Results:**

A total of 183 nodules were finally included; gender, nodule location, smoking history, and duration of follow‐up did not differ between the IAC group and the pre‐IAC group, whereas age was statistically different between the two groups. Of the 183 nodules, 52 showed growth and the predominant pathologic type was IAC, these IACs showed more PSN in nodule type, while the IAC group showed more significant differences in nodule type, nodules growth, and VDT than the pre‐IAC group. There were also differences in pathologic type and VDT between different Ki‐67 expression groups, and Ki‐67 expression gradually increased as VDT decreased.

**Conclusion:**

Lung adenocarcinoma (LUAD) presenting as GGNs exhibit distinct natural courses among pathologic subtypes. VDT effectively distinguishes these growth characteristics, with IACs showing shorter VDT. The significant correlation between VDT and Ki‐67 expression suggests that combining these parameters may provide valuable insights into the biological behavior and invasiveness of LUAD.

## 1 Introduction

Recent randomized controlled trials for lung cancer screening have demonstrated the efficacy of computed tomography (CT) in providing comprehensive histological imaging of lesions, also increasing detection rates for ground‐glass nodules (GGNs) [1]. Lung nodules refers to a small abnormal lesion in the lung, typically less than 3 cm in diameter, which may represent either benign or malignant conditions. While most pulmonary nodules are benign, some, especially GGNs, could indicate early‐stage lung cancer and therefore warrant close monitoring and further evaluation. Concurrently, lung nodule management guidelines have been published across various global regions [2, 3, 4, 5]. These guidelines recommend imaging follow‐up over several years based on nodule characteristics and growth patterns to assess for malignancy. If follow‐up imaging is suspicious for cancer, a multidisciplinary approach can facilitate further diagnostic interventions, greatly improving early diagnosis of lung cancer.

Persistent GGNs hold a high possibility of malignancy [6], Although the natural growth history of lung cancers with different degrees of invasiveness has not been clarified, volume doubling time (VDT) has been shown to stratify nodules based on their malignant potential. Nodules with a shorter VDT are generally associated with a poorer prognosis [7], suggesting that VDT may be an important predictor of tumor growth status. In addition, it has been found that nodules with smaller or no solid components tend to have longer VDT, indicating slower growth rates compared to those with larger solid components [8]. Therefore, combining the VDT with imaging features and further research on more reasonable follow‐up or treatment strategies can help to improve the prognosis of patients with early‐stage lung cancer.

The growth, invasion, and metastasis of malignant tumors is closely related to the proliferative state of tumor cells. Therefore, accurately evaluating the proliferative state of tumor cells is an important prerequisite for thoroughly investigating the biological behavior of lung malignancies. As a proliferation‐related protein expressed in the nucleus, Ki‐67 reflects the proliferative status of tumor cells and can be used to assess tumor malignancy. High Ki‐67 expression may indicate invasive and aggressive tumor cell growth [9] and is increasingly used to determine prognosis in cancer patients. Studies have demonstrated that high Ki‐67 expression has clinical value in predicting prognosis in lung adenocarcinoma (LUAD) [10].

Currently, there is insufficient data exploring correlations between GGNs growth patterns, pathologic features, and Ki‐67 expression. This study retrospectively analyzed clinical data of lung nodule patients who underwent prolonged follow‐up and eventual surgical resection at our hospital over the past several years. We aimed to investigate whether correlations exist between nodule natural course, pathologic features, and Ki‐67 expression in lung nodule patients. The goal is to provide more standardized, accurate, and reasonable follow‐up and treatment protocols for patients.

## 2 Materials and Methods

### 2.1 Research Method

We retrospectively reviewed the clinical data of patients who underwent surgical resection of lung nodules at Henan Provincial People's Hospital between December 2017 and October 2022. Patients who had undergone at least two CT scans at our hospital were screened and then further selected based on inclusion and exclusion criteria. Eligible cases were included in the study. Collected data consisted of general clinical information, original images of the last preoperative chest CT and previous CTs, imaging features of the nodules, and postoperative pathologic data. This retrospective study was approved by the Ethics Committee of Henan Provincial People's Hospital, and the personal privacy information of all patients was protected.

### 2.2 Inclusion and Exclusion Criteria

#### 2.2.1 Inclusion Criteria

(1) At least one lung nodule was found in lung CT. (2) Each patient has undergone surgical treatment, and the postoperative pathologic section is confirmed to be LUAD or precursor glandular lesions according to World Health Organization (WHO) lung tumor tissue classification review [11]. (3) At least two chest CT examinations were performed in each patient. (4) CT data should conform to the standard Digital Imaging and Communications in Medicine (DICOM) format.

#### 2.2.2 Exclusion Criteria

(1) Patients had undergone systemic chemotherapy and systemic anti‐tumor therapy. (2) The patient does not have clear pathologic results. (3) The patient's CT data does not meet the standard DICOM format of our hospital. (4) The patient has been diagnosed with other malignant tumors in the past.

### 2.3 Measurement of Ki‐67 Expression

All Ki‐67 assays were detected by immunohistochemistry and completed under the guidance of the Department of Pathology of our hospital. Immunohistochemical staining kit was purchased from Beijing Dingguo Changsheng Biotechnology Co. Ltd. and the detection method was to add mouse anti‐human Ki67 to the tissue slices after repairing them with 3% H_2_O_2_ and incubate them at 37°C for 30 min. rinse them with PBS for 3 times and then add the secondary antibody and incubate them at 37°C for 30 min, and then develop the color by DAB. There were known positive slices and negative controls, and HE staining was used as histological control. The relevant operations were carried out in strict accordance with the instructions of the kit.

### 2.4 Pathologic Diagnostic Criteria

Pathologic diagnosis needs to follow the criteria of the WHO Histologic Classification of Lung Tumors (5th edition, 2021), including adenomatous atypical hyperplasiar (AAH), adenocarcinoma in situ (AIS), minimally invasive adenocarcinoma (MIA) and invasive adenocarcinoma (IAC).

### 2.5 Imaging Methods

CT scanning was performed using CT machines such as Siemens SOMATOM Definition Flash and Philips Ingenuity in our hospital. Before scanning, patients were trained to breathe, patients were placed in the supine position, head first, and were scanned using deep inspiration followed by breath holding, the scanning range was from the lung tip to the top of the adrenal glands, and the two sides included the chest wall and axillary fossa.

### 2.6 Segmentation Effect Quality Control

All lung nodules were processed using the computer‐aided diagnosis (CAD) processing system. The segmentation effect was judged by two imaging physicians with more than 3 years of experience in reading films on the segmentation of nodules by the CAD system, and when there was disagreement, a third imaging physician assisted in the judgment. The two judges were required to correct the outline of the nodule layer by layer to ensure the accuracy of the measurement, and try to avoid the blood vessels and bronchial tubes as much as possible to ensure the accurate segmentation of the nodule.

### 2.7 Quantitative Definition of Lung Nodules

Lung nodule growth was defined as (1) an increase in the maximum diameter of any nodule by ≥ 2 mm [4]; (2) the appearance of any new solid component in the Pure Ground‐Glass Nodule (pGGN) [5].

VDT: refers to the time required to double the volume, which is used to describe the speed of growth of the observed subjects.VDT was calculated as follows:VDT = (LN(2) × T)/LN(X_f_/X_i_) (T is the time between two CT follow‐ups, X_f_ is the nodule volume at the latest follow‐up, and X_i_ is the nodule volume at baseline).

### 2.8 Statistical Methods

Statistical analysis was performed using SPSS 26.0 and RStudio 4.2.2 software. Normally distributed measurement data were expressed as mean ± standard deviation (M ± SD) and compared between groups using one‐way ANOVA. Non‐normally distributed measurement data were expressed as median (interquartile range) M(Q1, Q3) and compared using the Kruskal‐Wallis H or Mann–Whitney U tests. Enumeration data were expressed as example (percentage) and compared between groups using the Chi‐square test. Spearman correlation analysis was further used to calculate the correlation between Ki‐67 expression levels and VDT.

## 3 Result

### 3.1 General Clinical Features

According to the inclusion and exclusion criteria, a total of 183 nodules were finally included, of which there were 77 males (42.62%) and 106 females (57.38%), with an average age of 54.43 ± 12.46 years. There were 38 smokers (20.77%) and 145 non‐smokers (79.23%). Based on histopathologic results, nodules were classified as follows: adenocarcinoma precursor lesions in 37 cases (20.22%) (including 11 cases of AAH, 6.01%; 25 cases of AIS, 14.21%), MIA in 67 cases (34.43%), and IAC in 80 cases (45.36%). By lesion location, 63 cases (33.88%) were in the right upper lobe (RUL), 17 cases (9.29%) in the right middle lobe (RML), 25 cases (13.66%) in the right lower lobe (RLL), 53 cases (23.50%) in the left upper lobe (LUL), and 25 cases (14.21%) in the left lower lobe (LLL). Our study found that age was statistically different between the IAC and pre‐IAC groups (*p* < 0.05), while phase difference, smoking history, nodule location, and follow‐up time were not significantly different between the IAC and pre‐IAC groups (*p* > 0.05) (Table 1).

**TABLE 1:** Clinical characteristics with IAC and pre‐IAC.

Characteristic	Pre‐IAC group, (*n* = 103)	IAC group, (*n* = 80)	*p*
Age (year)	52.24 ± 12.75	57.24 ± 11.54	< 0.01^a^
Gender			
Male	40 (38.83)	37 (46.25)	0.313^b^
Female	63 (61.17)	43 (53.75)	
Smoking history			
Yes	20 (19.42)	18 (22.50)	0.683^b^
No	83 (80.58)	62 (77.50)	
Lesion location			
RUL	36 (34.95)	27 (33.75)	0.844^b^
RML	11 (10.68)	6 (7.50)	
RLL	12 (11.65)	13 (16.25)	
LUL	29 (28.16)	24 (30.00)	
LLL	15 (14.56)	10 (12.50)	
Follow‐up time (day)	219.00 (91.00, 475.00)	295.00 (80.50, 727.75)	0.335^c^

^a^ Independent sample *t* test.

^b^ Chi‐squared test.

^c^ Mann–Whitney *U* test.

### 3.2 Nodule Natural Course

Of the 183 nodules, 122 pGGN (63.93%), 19 heterogeneous ground‐glass nodule (hGGN) (36.07%), and 42 partially solid nodule (PSN) were detected at the time of initial detection of lung nodules, and 119 pGGN, 11 hGGN, and 53 PSN were detected in the 183 lung nodules after follow‐up. Fifty two nodules demonstrated growth, 131 had no significant change, and the growing lung nodules included 34 IAC, 16 MIA, only 2 AIS, and no growing AAH. Of the total growing nodules, 22 initially presented as pGGNs, 13 as hGGNs, and 17 as PSNs. Among the 22 pGGNs, 2 grew into PSNs, 1 into an hGGN, and 19 remained pGGNs. Of the 13 growing hGGNs, 10 progressed to PSNs, with only 3 remaining hGGNs. Notably, among the 52 growing lung nodules, the 34 IAC presented finally as PSNs in 25 cases, hGGNs in 2 cases, and pGGNs in 7 cases. Of the 18 pre‐IAC, 3 presented finally as PSNs, 2 as hGGNs, and 13 as pGGNs. These results suggest that the IAC group was more likely to appearance of solid component than the pre‐IAC group (*p* < 0.01). In addition, both the initial and final maximum diameters were larger in the IAC group than in the pre‐IAC group, with a more obvious increase in diameter. These findings indicate growing GGNs were more frequently IAC (*p* < 0.001) (Table 2).

**TABLE 2:** Growth of nodules with IAC and pre‐IAC.

Variable	Pre‐IAC group (*n* = 103)	IAC group (*n* = 80)	*p*
Nodule growth			
Yes	18 (17.48)	34 (42.50)	< 0.001^a^
No	85 (82.52)	46 (57.50)	
Initial nodule type			
Pggn	89 (86.41)	33 (41.25)	< 0.001^a^
Hggn	8 (7.77)	11 (13.75)	
PSN	6 (5.83)	36 (45.00)	
Final nodule type			
Pggn	89 (86.41)	30 (37.50)	< 0.001^a^
Hggn	6 (5.83)	5 (6.25)	
PSN	8 (7.77)	45 (56.25)	
Appearance of solid component			
Yes	2	9	< 0.01^a^
No	101	71	
Initial nodule diameter (mm)	6.91 (5.43,8.63)	11.85 (9.08,15.00)	< 0.001^b^
Final nodule diameter (mm)	7.19 (5.63,9.35)	13.65 (10.15,17.10)	< 0.001^b^
Maximum diameter increase			
Yes	17	31	< 0.001^a^
No	86	49	

*Note:* We also calculated differences in VDT between the two groups with different imaging presentations, finding a statistically significant difference between invasive and pre‐invasive adenocarcinomas, suggesting more rapid growth for IAC (*p* < 0.001). In addition, we found that tumor growth progressively accelerated as the solid component increased (Table 3).

^a^ Chi‐squared test.

^b^ Mann–Whitney *U* test.

**TABLE 3:** Different nodule types of VDT with IAC and pre‐IAC.

	Variable	Pre‐IAC group	IAC group	*p*
VDT	Overall	1902.00 (1195.00, 3413.00)	1097.00 (770.75, 1615.00)	< 0.001^a^
	Initial nodule type			
	Pggn	2209.00 (1202.50, 3695.50)	1392.00 (809.00, 1891.00)	< 0.001^a^
	Hggn	1830.00 (1315.25, 2570.75)	1252.00 (733.00, 1748.00)	0.137^a^
	PSN	1256.50 (968.75, 1729.75)	945.00 (577.50, 1246.00)	0.067^a^
	Final nodule type			
	Pggn	2209.00 (1202.50, 3695.50)	1388.00 (816.00, 2802.90)	< 0.001^a^
	Hggn	1708.50 (1066.00, 2149.50)	1983.00 (1693.50, 2695.00)	0.273^a^
	PSN	1496.50 (1064.00, 1909.75)	953.00 (583.00, 1253.50)	< 0.01^a^

^a^ Mann–Whitney *U* test.

### 3.3 Differences in Pathologic Types and Growth Between Different Ki‐67 Expression

Among the 183 lung nodules, Ki‐67 expression levels were measured in 92 nodules, including precursor glandular lesions in 8 cases (8.25%), with 2 AAH (2.06%), 6 AIS (6.19%), 37 MIA (38.14%), and 47 IAC (53.61%). These 92 nodules were divided into low and high expression groups based on a Ki‐67 cut‐off of 5%, the cut‐off value is bounded by the median of all assays for ki‐67 expression. Statistical results showed differences in pathologic types and VDT between the two groups, which were statistically significant (*p* < 0.05), while no statistical difference was found in nodule growth (*p* > 0.05) (Table 4).

**TABLE 4:** Pathologic, growth, and VDT of nodules with high and low ki‐67 expression.

Variable	Low ki‐67 expression (*n* = 49)	High ki‐67 expression (*n* = 43)	*p*
Pathologic types			
AAH/AIS MIA IAC	7 (13.73) 28 (52.94) 14 (33.33)	1 (2.17) 9 (21.74) 33 (76.09)	< 0.001^a^
Nodule growth			
Yes No	12 (13.73) 37 (13.73)	17 (13.73) 26 (13.73)	0.121^a^
VDT (day)	1720.00 (1061.00, 2476.00)	1060.00 (774.00, 1510.00)	< 0.01^b^

^a^ Chi‐squared test.

^b^ Mann–Whitney *U* test.

### 3.4 Correlation Between Ki‐67 Expression and VDT

**FIGURE 1:**
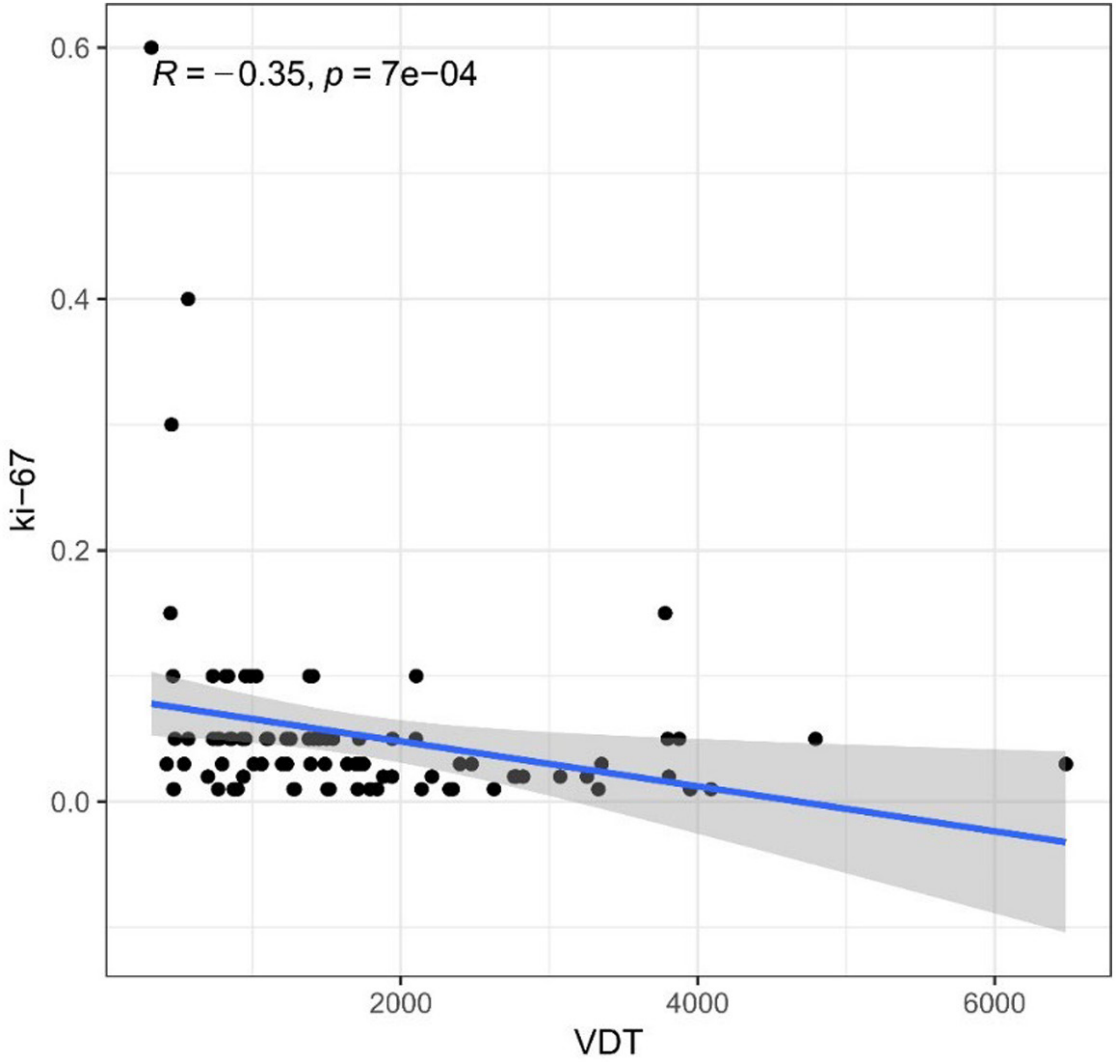
Correlation between VDT and Ki‐67 expression, and *R* was the correlation coefficient.

We further analyzed the correlation between VDT and Ki‐67 expression levels using Spearman's correlation. As shown in Figure 1, we found that VDT was negatively correlated with Ki‐67 expression, meaning that lower VDT was associated with higher Ki‐67 expression (*p* < 0.001) (Figure 1).

## 4 Discussion

Lung cancer is the most common malignancy worldwide and the leading cause of cancer‐related mortality, with increasing incidence and mortality rates globally in recent years. Approximately 75% of lung cancer patients are diagnosed at an advanced or metastatic stage, often with poor prognosis and low survival rates [12]. However, recent studies show 10‐year relapse‐free survival for AIS/MIA after surgery [13]. Therefore, early characterization of lung cancer invasiveness through lung nodule natural course is important for patient treatment and prognosis.

Growth of solid components often indicates malignant potential, and as early lung cancer invasiveness increases, tumor cell growth speeds up, manifesting as decreased VDT on imaging. In this study, IAC more frequently appeared as PSNs and showed greater enlargement during follow‐up compared to non‐invasive adenocarcinomas. Additionally, we found differences in VDT between pathologic types, with a median VDT of 1097 days in the IAC group versus 1902 days in the pre‐IAC group, indicating indolent growth. This aligns with previous studies showing indolent [14]. observed median VDTs of 1430.9 days for AIS, 1182.6 days for MIA, and 993.1 days for IAC among 29 pathologically confirmed GGNs. In a study of 51 pGGN LUAD using computer‐aided software, Qi. et al. [15] found median VDTs of 847.07 days for IAC and 4460.09 days for pre‐IAC. These findings demonstrate the slow growth of GGNs, allowing follow‐up rather than immediate resection, which provides time to exclude some benign lesions. At the same time, with the development of respiratory interventional technology, more and more new technologies such as hot steam ablation, microwave ablation, cryoablation, stereotactic body radiation therapy (SBRT), etc. have entered the clinic and are slowly maturing, and the inert growth of GGNs also provides time for the maturity of new technologies.

Additionally, while we can determine if GGNs are growing based on imaging changes, our study found that VDT showed better discrimination than growth assessed by imaging changes when using a Ki‐67 cut‐off of 5%. Currently, VDT has been incorporated into lung nodule management guidelines for several lung cancer screening trials [16, 17, 18]. In lung cancer screening, overdiagnosis and overtreatment are important considerations. Studies estimate 20%–25% of screen‐detected lung cancers may be overdiagnosed [19], as these indolent tumors are unlikely to result in mortality despite diagnosis. VDT is now considered a suitable tool to distinguish indolent from invasive tumors [20], although some limitations exist including segmentation effects, follow‐up duration, CT machine performance, and parameter settings [7]. However, compared to conventional 2D assessment, VDT shows superior performance.

Ki‐67 is a nuclear proliferation protein representing tumor cell proliferative activity and is highly valuable for diagnosis, treatment, and prognosis of lung cancer patients. Current research shows Ki‐67 expression is significantly higher in malignant tumors compared to normal tissues [21, 22] and tends to increase with decreasing tissue differentiation. Ki‐67 correlates closely with occult metastasis and clinical tumor stage [23, 24]. When used appropriately in conjunction with known pathologic characteristics of malignancies, Ki‐67 can be a reliable indicator of tumor recurrence risk [25]. Numerous studies demonstrate associations between Ki‐67 expression and patient prognosis across various cancers [26, 27, 28]. Thus, the importance of Ki‐67 expression for pre‐treatment assessment of tumor invasiveness and selection of appropriate therapies is increasingly recognized. In this study, Ki‐67 expression correlated with VDT. After comprehensive evaluation of suspicious lung nodules, Ki‐67 could be used as an additional marker to predict patient prognosis based on nodule VDT.

It should be noted that this was a retrospective analysis, and the included nodules were primarily more malignant SSNs with surgical indications, leading to potential selection bias. Additionally, we did not further investigate distinguishing benign versus malignant nodules appearing as GGNs. To address these limitations, a prospective, multicenter study with a larger sample size is warranted to thoroughly characterize nodule features, elucidate natural course of GGNs, and provide more robust evidence to inform clinical decision making and patient management.

## 5 Conclusion

Our study found that early‐stage lung cancers appearing as GGNs demonstrate differential natural course among pathologic subtypes. VDT serves as an effective tool to distinguish these variable growth characteristics. Additionally, we found a significant negative correlation between VDT and Ki‐67 expression, with increasing Ki‐67 levels associated with decreasing VDT.

## Author Contributions

**Shaohui Huang:** writing – review and editing (lead). **Huanhuan Zhou:** formal analysis (equal). **Chenchen Lin:** formal analysis (equal). **Ziqi Wang:** data curation (equal). **Lijun Shen:** data curation (equal). **Ya Sun:** formal analysis (equal). **Meihui Wei:** data curation (equal). **Zhiwei Xu:** data curation (equal). **Xiaoju Zhang:** project administration (lead).

## Ethics Statement

The study was ethically approved by the Ethics Committee of Henan Provincial People's Hospital.

## Consent

As this study was retrospective, no informed consent was required from patients or legal representatives.

## Conflicts of Interest

The authors declare no conflicts of interest.

## Data Availability

The data sets used and/or analyzed during the current study are available from the corresponding authors on reasonable request.
